# Nurses Wanted

**Published:** 1918-10

**Authors:** 


### Nurses Wanted.

"The United States government has placed those student nurses in the hands of the state divisions. Each county and local unit of the woman's committee in Texas is asked to provide for the establishment of recruiting stations, or places where enrollment in the United States student nurse reserve can be made.

"The call is for women of sound health, between the ages of 19 and 35, having full high school training wherever possible, and preferably college training.

"The campaign is to be closed on the evening of Aug. 11, unless it be necessary to continue the work longer.

"There are several nurses' training schools in Texas. State representative of the national organization for public health nursing is Mrs. Ethel S. Parsons, division of health, San Antonio.

"Women will be given an opportunity to enroll in any one of three ways:

"1. As engaging to hold themselves in readiness until April 1, 1919, to accept assignments to nurses' training schools in civilian hospitals.

"2. As desiring to become candidates for the army nursing school recently established by authority of the war department with branch schools in selected military hospitals.

"3. As engaging to hold themselves in readiness until April 1, 1919, to accept assignments to either a civilian training school or the army nursing school. Those who so enroll will be called where the first need arises. The government hopes that a majority of those who enroll will thus put down their names for both.

"The enrollment card will indicate two classes of registrants, preferred and deferred. The preferred class will be those who are ready to accept assignment to whatever hospital the government directs them. Those who register in the preferred class will be assigned first.

"The United States student nurse reserve is the equivalent for women, that the great national army training camps are for soldiers."

